# Familial Risks and Estrogen Receptor-Positive Breast Cancer in Hong Kong Chinese Women

**DOI:** 10.1371/journal.pone.0120741

**Published:** 2015-03-10

**Authors:** Lap Ah Tse, Mengjie Li, Wing-cheong Chan, Chi-hei Kwok, Siu-lan Leung, Cherry Wu, Ignatius Tak-sun Yu, Wai-cho Yu, Xiangqian Lao, Xiaorong Wang, Carmen Ka-man Wong, Priscilla Ming-yi Lee, Feng Wang, Xiaohong Rose Yang

**Affiliations:** 1 JC School of Public Health and Primary Care, the Chinese University of Hong Kong, Hong Kong SAR, China; 2 Department of Surgery, North District Hospital, Hong Kong SAR, China; 3 Department of Oncology, Princess Margaret Hospital, Hong Kong SAR, China; 4 Department of Surgery, Pamela Youde Nethersole Eastern Hospital, Hong Kong SAR, China; 5 Department of Pathology, North District Hospital, Hong Kong SAR, China; 6 Department of Medicine, Princess Margaret Hospital, Hong Kong SAR, China; 7 Genetic Epidemiology Branch, Division of Cancer Epidemiology & Genetics, National Cancer Institute, National Institutes of Health, Bethesda, United States of America; MOE Key Laboratory of Environment and Health, School of Public Health, Tongji Medical College, Huazhong University of Science and Technology, CHINA

## Abstract

**Purpose:**

The role of family history to the risk of breast cancer was analyzed by incorporating menopausal status in Hong Kong Chinese women, with a particular respect to the estrogen receptor-positive (ER+) type.

**Methods:**

Seven hundred and forty seven breast cancer incident cases and 781 hospital controls who had completed information on family cancer history in first-degree relatives (nature father, mother, and siblings) were recruited. Odds ratio for breast cancer were calculated by unconditional multiple logistic regression, stratified by menopausal status (a surrogate of endogenous female sex hormone level and age) and type of relative affected with the disease. Further subgroup analysis by tumor type according to ER status was investigated.

**Results:**

Altogether 52 (6.96%) breast cancer cases and 23 (2.95%) controls was found that the patients’ one or more first-degree relatives had a history of breast cancer, showing an adjusted odds ratio (OR) of 2.41 (95%CI: 1.45–4.02). An excess risk of breast cancer was restricted to the ER+ tumor (OR = 2.43, 95% CI: 1.38–4.28), with a relatively higher risk associated with an affected mother (OR = 3.97, 95%CI: 1.46–10.79) than an affected sister (OR = 2.06, 95%CI: 1.07–3.97), while the relative risk was more prominent in the subgroup of pre-menopausal women. Compared with the breast cancer overall, the familial risks to the ER+ tumor increased progressively with the number of affected first-degree relatives.

**Conclusions:**

This study provides new insights on a relationship between family breast cancer history, menopausal status, and the ER+ breast cancer. A separate risk prediction model for ER+ tumor in Asian population is desired.

## Introduction

Breast cancer is one of the most commonly diagnosed cancer worldwide, and it is the third leading cause of cancer death in Hong Kong women[1,2]. Although Hong Kong is still considered as a low-risk region with breast cancer incidence rate 3-fold lower than that of the United States[1], the age-standardized incidence rate has almost doubled from 1983 (35.7 per 100,000) to 2011 (61.0 per 100,000) with an accelerating rate for women aged below 50 years[2]. Genetic susceptibility, westernization of lifestyle and other environmental exposure are likely to have contributed to the etiology of breast cancer in Hong Kong women[3]. Evidence consistently showed that family history of breast cancer in the first-degree relatives (FDR) was associated with 110% (95% confidence interval [95% CI]: 100–120%) excess risk of breast cancer compared with those without a family history[4]; this association, however, might not be entirely attributable to the genetic variations (e.g., BRCA1/2 mutations) and the contributions from shared environmental factors, such as a prolonged exposure to endogenous and exogenous female sex hormones in a familial microenvironment, could have played a role[5].

Breast cancer is a heterogeneous disease that can be divided into distinct tumor subtypes commonly classified by the status of estrogen receptor. Clinically, estrogen receptor-positive (ER+) breast cancer has better responsiveness to antiestrogens compared to ER-negative (ER-) subtype[6]. Evidences from a number of epidemiologic studies also suggested that established risk factors might have differential effects on the risk of ER+ and ER- breast cancer. In particular, reproductive risk factors appeared to be more strongly linked to ER+ or progesterone receptor-positive (PR+) cancers than to receptor negative tumors[6,7]. These relationships are compatible with the probable importance of cumulative exposure to sex-steroid hormones in the pathogenesis of ER-positive breast cancer[8]. Only a few studies assessed the role of family history in the etiology of breast cancer according to ER status and menopausal status together[9].It is generally believed that pre- and post-menopausal breast cancer may be two different diseases that are not only driven by hormone difference but also by age. In this study, the role of family history to the risk of breast cancer was analyzed by incorporating menopausal status (as a surrogate of endogenous female sex hormone level and age) in Hong Kong Chinese women, with a particular respect to the ER+ tumor type.

## Materials and Methods

The study protocol was approved by both the Joint Chinese University of Hong Kong-New Territories East Cluster Clinical Research Ethics Committees and the Kowloon West Cluster. The informed consent for both cases and controls was obtained before the interview. Chinese women, aged between 20 and 84 with newly diagnosed primary breast cancer (ICD-10 code 50), were confirmed by histology. All eligible cases were consecutively identified from the Department of Surgery or Clinical Oncology of 3 hospitals in Hong Kong during the period 11/2011 to 05/2014, and were interviewed within 3 months after diagnosis. Seventy hundred and sixty seven eligible cases were interviewed and the response rate was 91.1%, and the main reasons for the non-responses were no interest and poor medical condition. Each breast cancer incident case was frequency matched in 5-year age groups by a control patient selected from the same hospital where the cases came from. We recruited 792 eligible controls with a broad disease pattern of diagnosis, with the response rate of 93%. To be eligible, the cases and controls must have no previously physician-diagnosed cancer at any site.

Personal interviews were conducted by trained interviewers using a standardized questionnaire to obtain information on age at diagnosis (or age at interview for the controls), educational attainment, smoking habits, alcohol drinking, body mass index (BMI), history of benign breast diseases, family cancer history, occupational history, and reproductive factors including age at menarche, menopausal status, parity, breastfeeding, age at first birth and hormone replacement therapy. In terms of family cancer history, each participant was required to report whether their FDR had ever been diagnosed by a doctor of having cancer at any site. If a positive answer was obtained, further information on which family member and what type of cancer was diagnosed should be collected.

The data on ER status (for cases only) was extracted and the disease diagnosis for the cases and controls from the hospital medical records were verified. The quality of the data about family cancer history was assessed by re-interviewing 25% of the cases and controls about 6 weeks after the initial interviews, and the agreement between the two interviews was high (kappa = 0.62).

Unconditional multivariate logistic regression models was performed to estimate the adjusted odds ratio (OR) and 95% confidence intervals (CIs) for the association with each family cancer history variable after controlling for potential confounding variables that were statistically significant at p < 0.05 from the univariate analysis (**Table 1**). A case-case analysis was conducted to test the heterogeneity of family breast history in FDR by ER status. Further stratified analyses were conducted according to menopausal and ER status, while the gradient relationship of breast cancer risk with increasing number of FDR impacted was further explored by trend tests.

**Table 1:** Selected characteristics of breast cancer cases and controls among Hong Kong Chinese women, 2011–2014^a^.

Characteristics by Category	Cases		Controls		*P value* ^b^
	No.	%	No.	%	
Total No.	747	100	781	100	
Cigarette smoking					0.51
Yes	50	6.7	59	7.6	
No	697	93.3	722	92.4	
Alcohol drinking					0.43
Yes	31	4.1	39	5.0	
No	716	95.9	742	95.0	
Education					0.51
Primary school	280	37.5	280	35.9	
High school	341	45.6	374	47.9	
College or above	93	12.4	111	14.2	
Unknown	33	4.4	16	2.0	
Parity					<0.05
0	131	17.5	138	17.7	
1–2	419	56.1	404	51.7	
⩾3	178	23.8	232	29.7	
Unknown	19	2.5	7	0.9	
Ever use of oral contraceptives					0.26
Yes	252	33.7	288	36.9	
No	476	63.7	487	62.4	
Unknown	19	2.5	6	0.8	
Hormone replacement therapy					0.07
Yes	17	2.3	31	4.0	
No	713	95.4	744	95.3	
Unknown	17	2.3	6	0.8	
Menopausal status					0.56
Pre-menopausal	279	37.3	304	38.9	
Post-menopausal	446	59.7	470	60.2	
Unknown	22	2.9	7	0.9	
Estrogen receptor status^c^					
ER+	438	76.4			
ER−	135	23.6			
	Mean±SD		Mean±SD		
Age at interview (years)	55.68±11.7		53.47±11.7		<0.01
Age at menarche (years)	13.6±2.0		13.8±2.3		0.02
Age at first birth (years)	26.5±4.8		25.5±4.7		<0.01
Body mass index (kg/m^2^)	23.5±3.7		23.0±3.9		0.01

Abbreviation: ER, estrogen status; ER+, estrogen receptor-positive, ER−, estrogen receptor-negative.

^a^ Subjects with missing values were excluded from the analyses.

^b^ Chi-squared test for categorical variables and independent t test for continuous variables for the comparison of breast cancer cases and hospital controls.

^c^ There were 174 (23.3%) of breast cancer cases without a status of estrogen receptor.

## Results

Seven hundred and forty seven breast cancer cases and 781 controls had completed information on family cancer history (**Table 1**). Overall, breast cancer cases were significantly younger than the controls at the age of menarche, but they had a relatively higher proportion of hormone replacement therapy. Compared with breast cancer cases, the controls had more children, lower level of BMI, and younger age at first birth. The distribution of educational attainment, tobacco smoking, alcohol drinking, oral contraceptives usage, and menopausal status was similar between the cases and controls. ER+ tumor accounted for 76.4% (438/573) of breast cancer cases with a known status of estrogen receptor. Similar distribution of ER status appeared between cases <50 years (ER+ vs. ER-: 75.7% vs. 24.3%) and those aged 50 years or above (post-menopausal women) (ER+ vs. ER-: 76.8% vs. 23.2%) (p = 0.76).

A total of 277 (37.1%) breast cancer cases and 214 (27.4%) controls were reported a family history of any type of cancer in the FDR, showing an adjusted OR of 1.47 (95%CI: 1.17–1.83). This moderate association with breast cancer risk was mainly driven by a family cancer history of the head and neck (OR = 1.73, 95% CI: 1.00–3.00, 38 cases), genitourinary (OR = 1.91, 95% CI: 1.09–3.35, 38 cases), and the breast (OR = 2.41, 95% CI: 1.45–4.02, 52 cases). About 1.4 times more breast cancer cases than the controls having a history of breast cancer in mother or sister (6.96% vs. 2.95%). Compared with a sister impacted by breast cancer (OR = 1.90, 95% CI: 1.05–3.44) (**Table 2**), the OR was greater when the mother (3.90, 95% CI: 1.55–9.83) was impacted. Case-case analysis by ER status showed that slightly more cases with a family history of breast cancer in the FDR presented ER+ tumor than those without a family history, whilst the heterogeneity was not statistically significant (77.5% vs. 75.8%, p = 0.647) due to a small sample size in the ER- type; nevertheless, there was no obvious difference in the age at diagnosis of breast cancer between women with (56.04±9.60 years) and without a family history (55.44±12.80 years, p = 0.49).

**Table 2:** Odds ratios of breast cancer and by estrogen receptor (ER) status in Hong Kong Chinese women with a history of any cancer or breast cancer in the first-degree relatives.

Cancer history in the first-degree relatives	Cases	Controls	Adjusted OR (95% CI) ^a^
	(N = 747)	(N = 781)	
**Any cancer**	277	214	1.47 (1.17–1.83)
Parents	182	155	1.24 (0.97–1.60)
Siblings	131	81	1.69 (1.25–2.29)
**Breast cancer**			
*All cases*	52	23	2.41 (1.45–4.02)
Mother	22	6	3.90 (1.55–9.83)
Sister	33	18	1.90 (1.05–3.44)
*ER+ breast cancer*	31	23	2.43 (1.38–4.28)
Mother	13	6	3.97 (1.46–10.79)
Sister	21	18	2.06 (1.07–3.97)
*ER− breast cancer*	4	23	0.82 (0.27–2.45)
Mother	2	6	1.60 (0.31–8.16)
Sister	2	18	0.51 (0.12–2.29)

Abbreviation: OR, odds ratio; 95% CI, 95% confidence interval; ER+, estrogen receptor-positive, ER−, estrogen receptor-negative.

^a^ Adjusted for age at interview, age at menarche, age at first birth, parity, education attainment, hormone replacement therapy, and body mass index.

Subgroup analysis according to ER status showed that an excess risk of breast cancer was restricted to the ER+ cases, with an OR of 3.97 (95%CI: 1.46–10.79) for those with a mother impacted by breast cancer and 2.06 (95%CI: 1.07–3.97) for those who had an impacted sister. Stratified analysis by menopausal status demonstrates that a significantly increased risk of breast cancer was confined to the pre-menopausal women (indicating a higher female sex hormone level and younger age) who had mother impacted by breast cancer (OR = 5.39, 95% CI: 1.13–25.61), and this association was enhanced for women with the ER+ tumor type (OR = 8.67, 95%CI: 1.78–42.25). A moderate excess risk of breast cancer was also indicated in the post-menopausal women, but there was no statistical significance (**Table 3**). There was a similar pattern of the results when subgroup analysis was conducted according to the mean age at the diagnosis of breast cancer (55 years).

**Table 3:** Odds ratios of all breast cancers and the ER+ breast cancers in Hong Kong Chinese women with a history of breast cancer in first-degree relatives, stratified by menopausal status.

History of breast cancer in first degree relatives		Pre-menopausal			Post-menopausal	
	Cases	Controls	OR (95% CI)^a^	Cases	Controls	OR (95% CI)^a^
	(N = 280)	(N = 304)		(N = 467)	(N = 477)	
All breast cancers	21	9	2.25 (0.99–5.12)	31	14	2.33 (1.20–4.52)
Mother	10	2	5.39 (1.13–25.61)	12	4	2.88 (0.90–9.21)
Sister	12	7	1.55 (0.59–4.11)	21	11	2.04 (0.95–4.39)
ER + breast cancers	15	9	3.00 (1.26–7.15)	16	14	1.95 (0.90–4.22)
Mother	8	2	8.67 (1.78–42.25)	5	4	1.77 (0.45–6.92)
Sister	8	7	1.79 (0.61–5.23)	13	11	2.18 (0.92–5.14)

Abbreviation: OR, odds ratio; 95% CI, 95% confidence interval; ER+, estrogen receptor-positive, ER−, estrogen receptor-negative.

^a^ Adjusted for age at interview, age at menarche, age at first birth, parity, education attainment, hormone replacement therapy, and body mass index.

As shown in **Table 4**, an increased risk of breast cancer was associated with an increasing number of FDR who had a history of any cancer, while this trend tended to be stronger for the association with increasing number of the FDR impacted by breast cancer; these positive gradients were more prominent for the association with the ER+ breast cancer.

**Table 4:** Associations of numbers of first-degree relatives affected with any cancer or breast cancer with the risk of all breast cancer cases or the ER+ tumor type in Hong Kong Chinese women.

No. of first-degree relatives affected	Controls	All breast cancers		ER+ breast cancers	
	(N = 781)	(N = 747)	OR (95% CI)^a^	(N = 438)	OR (95% CI)^a^
Any cancer					
0	567	470	1.00	567	1.00
1	167	196	1.36 (1.07–1.74)	167	1.35 (1.01–1.80)
⩾2	47	81	1.83 (1.24–2.70)	47	1.96 (1.27–3.04)
*Test for trend*			*P<0.01*		*P<0.01*
Brest cancer					
0	758	695	1.00	758	1.00
1	21	44	2.27 (1.32–3.90)	21	2.21 (1.20–4.05)
⩾2	2	8	3.84 (0.80–18.55)	2	4.62 (0.89–23.86)
*Test for trend*			*P<0.01*		*P<0.01*

Abbreviation: OR, odds ratio; 95% CI, 95% confidence interval; ER+, estrogen receptor-positive, ER-, estrogen receptor-negative.

^a^ Adjusted for age at interview, age at menarche, age at first birth, parity, education attainment, hormone replacement therapy, and body mass index.

## Discussion

One merit of this study is that a more progressively increased risk of ER+ breast cancer was demonstrated with the additional FDR impacted by breast cancer in the pre-menopausal women that was never reported in the previous studies. A doubled risk of breast cancer in Hong Kong Chinese women with a history of breast cancer in the FDR were analyzed, and this result is in line with most original studies and a meta-analysis by pooling 74 breast cancer studies in which a positive association between breast cancer and family breast cancer history was reported[4,9,10]. Similar to the result of Shanghai women’s health study, a greater risk of breast cancer was demonstrated in the pre-menopausal women who reported a mother impacted by breast cancer (5.39) than an impacted sister (1.55), whereas the magnitude of association tends to be stronger than the Shanghai women's health study (2.35 vs.1.23)[10]; nevertheless, both the results of Shanghai and our studies did not support a homogeneous association of breast cancer risk with an affected mother or a sister that was reported in a collaborative reanalysis of 52 epidemiological studies[11]. We are aware that the Shanghai study included in the collaborative research also provided some indications that more mothers than sisters were affected with breast cancer[11,12].

Familial risks reflect not only shared genetic components but also environmental influencing factors, compassing any shared diets, lifestyles, reproductive factors, and familial microenvironment exposures. Previous studies consistently showed that approximate 10–15% of breast cancers have family history of the disease, but only 5% of them could be explained by the rare but highly penetrant mutations in BRCA1/2[13]. The majority of the unexplained fraction of familial risk is likely to be explained by a polygenic model implying a combination of many variants of a weak potency to the disease as well as shared environmental exposures[14–16]. A Nordic twin study showed that 73% of the variation of breast cancer among monozygotic and dizygotic twins could be explained by the environmental exposures[17]. Clustering of breast cancer cases may be a reflection of similar background of exposures to endogenous and exogenous female sex hormones that correlate between family members, such as reproductive risk factors (e.g., low parity, young age at menarche, late age at menopause) which have been estimated to account for approximate 15% of the overall risk[18].

Epidemiological studies revealed that the association of breast cancer with many susceptibility loci (e.g., FGFR2, TNRC9, ADAM29, 8q24, 2q35, and 5p12) were significantly modified by estrogen receptor status[19–23]; among them a variant in FGRF2, 5p12, and a SNP on 4q34.1/ADAM29 (in Chinese women) was evident to be linked to the ER+ tumor[20–22]. The observed differences in genetic associations by tumor subtypes tended to support that the ER+ and ER- tumors were resulted from different etiologic pathways. Although a greater risk of ER+ breast cancer was observed among our pre-menopausal women in particular among those who had an affected mother which indicates an importance of genetic contribution, the value of analyzed data was restricted to assess the extent to what the familial risks to ER+ breast cancer could be explained by the genetics. Further epidemiological studies involving adequate data on individual genes mutations and environmental exposures (shared and non-shared) across different generations are desired to address this knowledge gap.

Results from this hospital-based case-control study were regarded as valid and were expected to have general application. The breast cancer cases were recruited from several large hospitals, and the age and distribution of our breast cancer cases by histological subtypes were highly similar to those reported by the Hong Kong Cancer Registry. The response rate for breast cancer cases was high (92%) and the misclassification of breast cancer status was minimal as all cases included were confirmed by histology. Despite population controls were theoretically preferred to hospital controls in a case-control study[24], there were practical issues particularly a low response rate (40% or even below according to our experiences) that might largely damage the generalization of the results. Another concern with population controls was the potential for the recall accuracy not being comparable with the cases, whilst the diseased controls had comparable quality of information acquired to that of the cases especially for a family disease history, which in turn minimized the recall and interview bias. We recruited hospital controls with a broad differential diagnosis that were unrelated to the exposure of interest (i.e., family cancer history), and these controls are thus appropriate[25].

Data from a special group of 117 hospital controls (who underwent biopsy for suspected breast cancer and were handled as breast cancer cases during the interviews, but eventually were confirmed to be the non-cancer cases) showed a lower proportion of a family history of breast cancer (6% vs. 7.0%) or any cancer (31.6% vs. 37.1%) in the FDR than the surgically confirmed breast cancer. The differential family cancer history reported in these 2 subgroups suggested the lack of interviewer bias and, hence, provided further support for the validity of our results. Another concern is a potentially increased surveillance of breast cancer among women with a family history of the disease, which may lead this group of women to presenting less advanced staging of breast cancer than those without a family history. Little difference in early breast cancer (e.g., staging I) was noticed between women with and without a family history (11.5% vs. 10.6%) (Data not shown). The population attributable risk was estimated because the breast cancer cases in our study was a reasonable representative to the general population, and was found that about 4.1% and 11.8% of incident breast cancer cases could be attributable to having a family history of breast cancer or any cancer in FDR[26]. Nevertheless, the observed odds ratios, particularly for the cases with ER-, were not statistically significant with rather broad 95% confidence intervals, indicating the lack of adequate sample size to study the association with family history. Further studies with more breast cancer cases and adequate numbers for different tumor types of breast cancer are warranted.

In conclusion, this study provided new insights on a relationship between family breast cancer history, menopausal status, and the ER+ breast cancer. An establishment of an individual risk factor model for ER+ and ER- breast cancer is desired for Asian population and this may advance the current Gail model in which no separation of risk prediction model was provided.
